# Supplementary material for: Quantitative SARS-CoV-2 Spike Antibody Response in COVID-19 Patients Using Three Fully Automated Immunoassays and a Surrogate Virus Neutralization Test
Source: Diagnostics (Basel). 2021 Aug 19;11(8):1496. doi: 10.3390/diagnostics11081496 (PMC8393767; doi:10.3390/diagnostics11081496)
Supplement: Supplementary file 1 [file diagnostics-11-01496-s001.zip › diagnostics-1327236-SI.pdf]

**Supplementary Materials**

**Table S1.** Comparisons of SARS-CoV-2 S protein antibody levels by three chemiluminescent immunoassays (Abbott, Roche and Siemens) and the surrogate virus neutralization test (Genscript) in COVID-19 patients according to days after symptom onset and disease severity

| | | Days after symptom onset | | | | | |
|---|---|---|---|---|---|---|---|
| | Severity | ≤5 | 6-8 | 9-11 | 12-14 | 15-21 | 22≤ |
| Abbot (AU/mL) | severe or critical | 5.1† (1.7-231.1) | 17.6 (3.9-2117.0) | 1128.4 (256.4-6656.0) | 4670.2* (1184.2-7892.3) | 7621.6* (3900.6-11323.4) | 6615.8* (3855.8-7856.7) |
| | mild | 6.5 (0.9-87.3) | 13.1 (3.7-128.4) | 95.5 (25.5-722.7) | 1247.6 (181.1-2628.1) | 955.7 (162.7-3573.2) | 3002.6 (309.0-6552.7) |
| Roche (U/mL) | severe or critical | 0.4 (0.4-4.5) | 0.4 (0.4-49.3) | 11.7 (2.1-130.5) | 72.9 (19.6-160.5) | 184.0 (148.3-386.4) | 199.5 (129.1-378.6) |
| | mild | 0.4 (0.4-0.9) | 0.5 (0.4-14.2) | 4.1 (0.5-43.6) | 62.6 (3.0-101.7) | 64.7 (2.8-130.6) | 89.7 (7.9-715.9) |
| Siemens (U/mL) | severe or critical | 0.1 (0.0-1.6) | 0.2 (0.0-12.5) | 7.2* (1.9-45.2) | 31.1* (9.4-53.5) | 72.8* (36.9-95.5) | 49.5* (34.7-95.7) |
| | mild | 0.1 (0.0-0.9) | 0.2 (0.0-0.8) | 1.1 (0.1-4.9) | 7.4 (1.1-22.5) | 8.5 (0.9-22.8) | 19.1 (1.7-61.2) |
| Genscript (%) | severe or critical | 11.0 (3.0-42.5) | 28.0 (7.4-78.5) | 71.0 (47.8-87.3) | 90.0 (65.3-93.0) | 92.0 (86.9-95.0) | 88.5 (85.0-95.0) |
| | mild | 13.0 (8.2-19.8) | 13.0 (2.5-59.4) | 40.0 (17.3-80.1) | 82.5 (31.5-90.1) | 82.0 (36.1-92.2) | 89.5 (47.2-94.8) |

*Severe or critical vs. mild ($p < 0.05$). †Median (95% confidence interval)

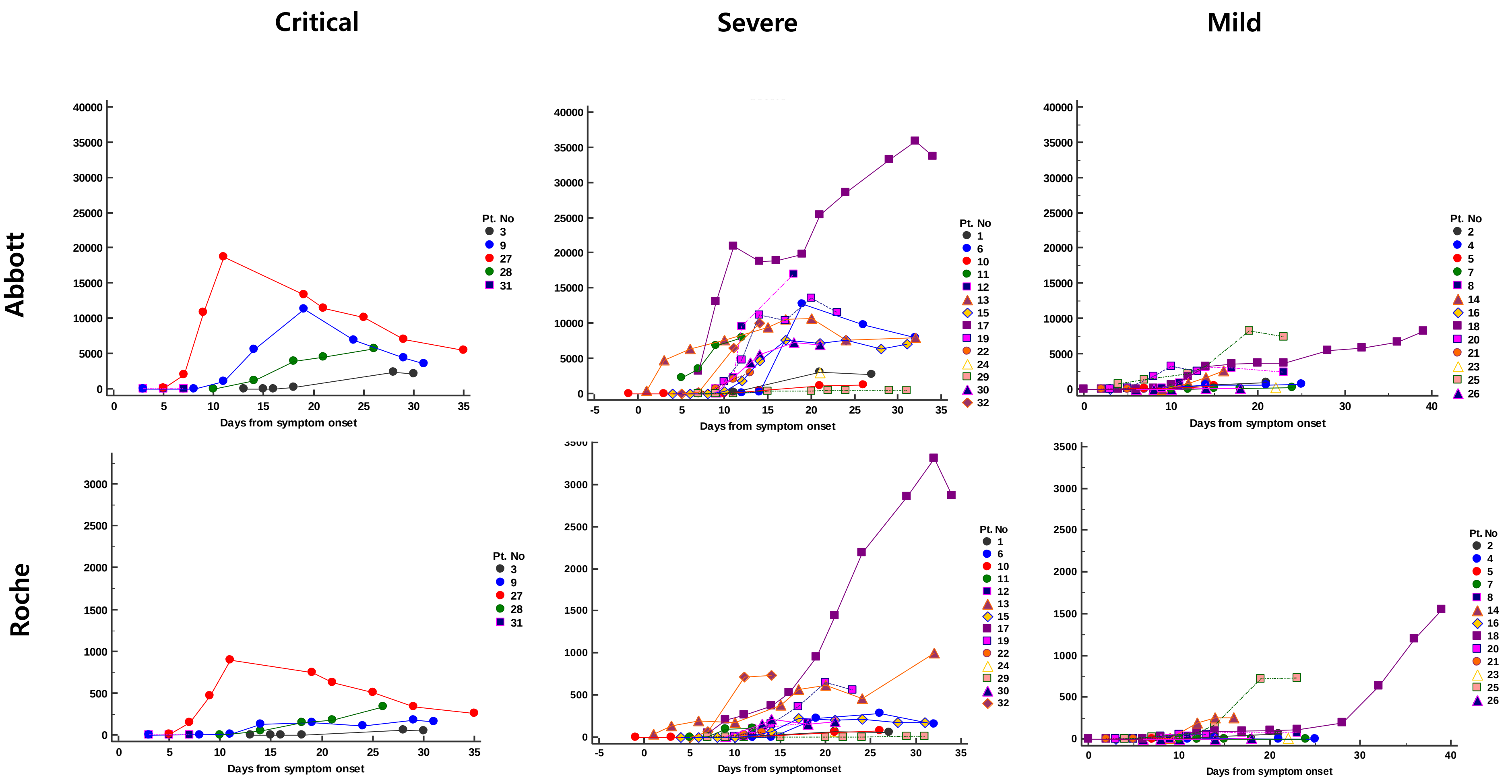
Critical
Severe
Mild
Abbott
Roche
Pt. No
3
9
27
28
31
Pt. No
1
6
10
11
12
13
15
17
19
22
24
29
30
32
Pt. No
2
4
5
7
8
14
16
18
20
21
23
25
26
Days from symptom onset
Days from symptomonset

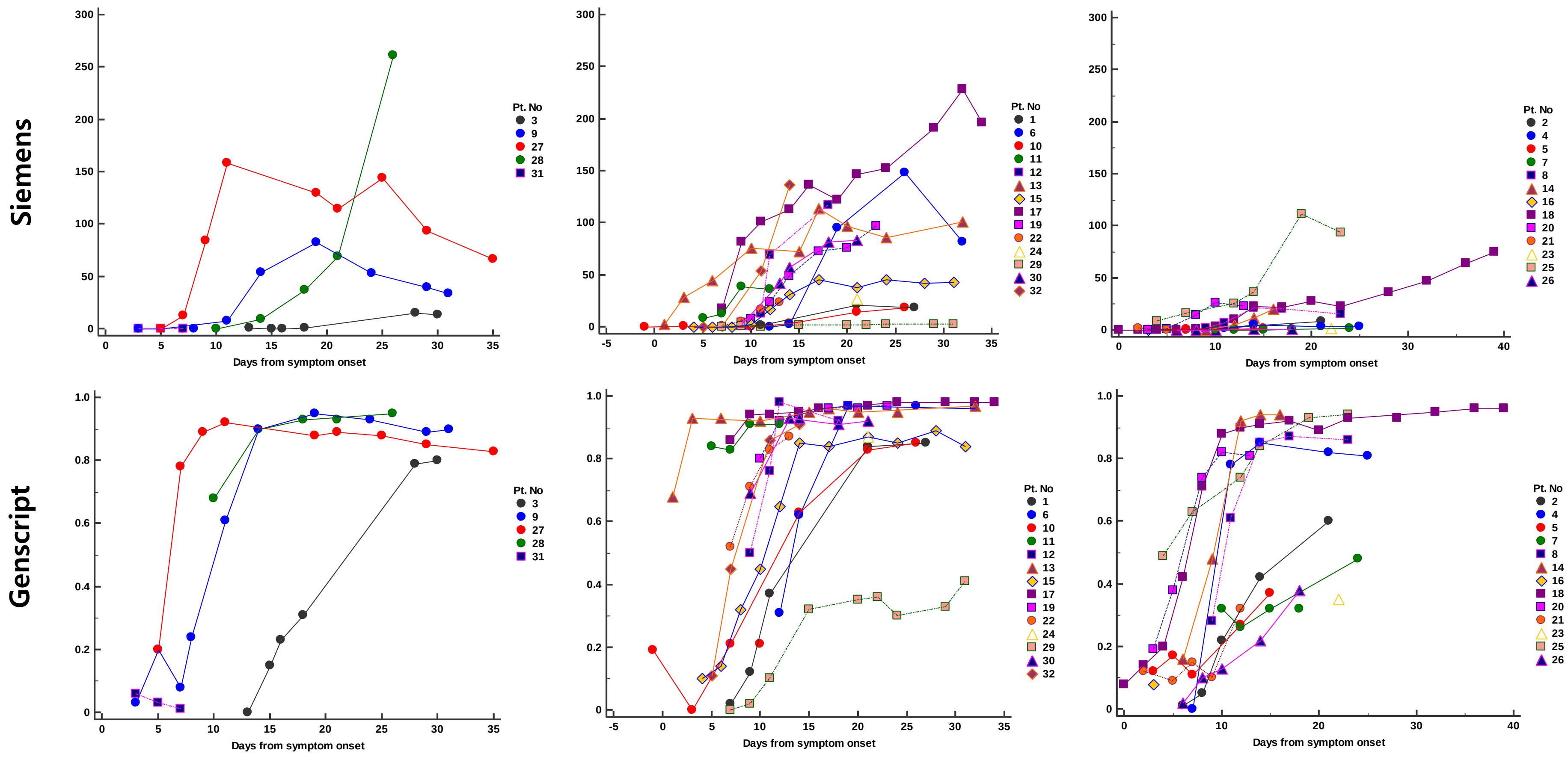


**Figure S1.** SARS-CoV-2 S protein antibody levels measured by three chemiluminescent immunoassays and the surrogate virus neutralization test in 32 COVID-19 patients. Antibody levels are plotted according to days after symptom onset and disease course (critical, severe, or mild) in each assay.

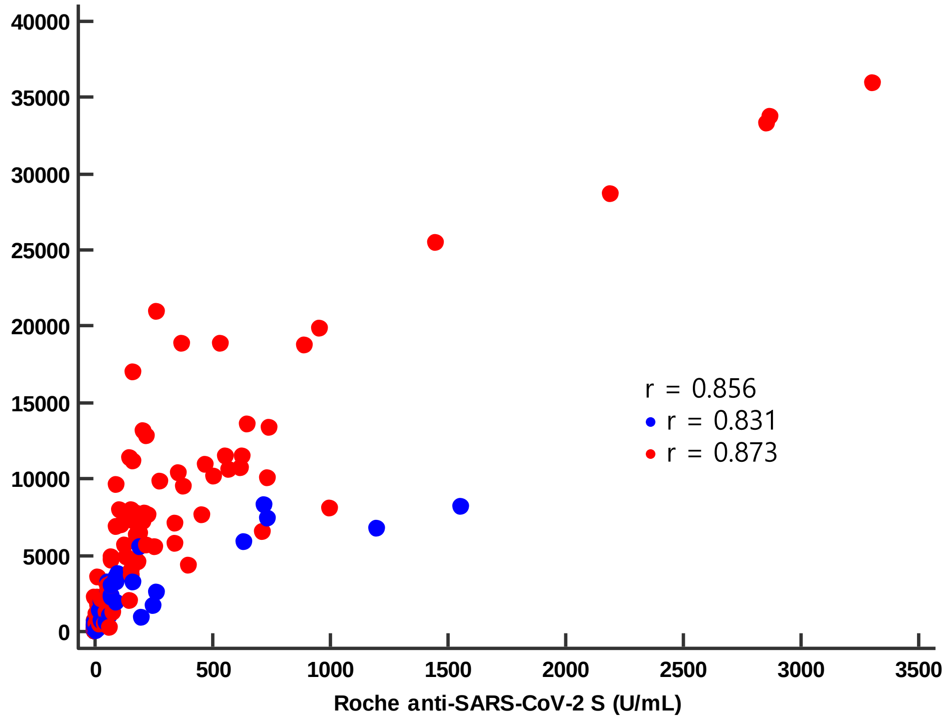
40000
35000
30000
25000
20000
15000
10000
5000
0
0
500
1000
1500
2000
2500
3000
3500
Roche anti-SARS-CoV-2 S (U/mL)
r = 0.856
r = 0.831
r = 0.873

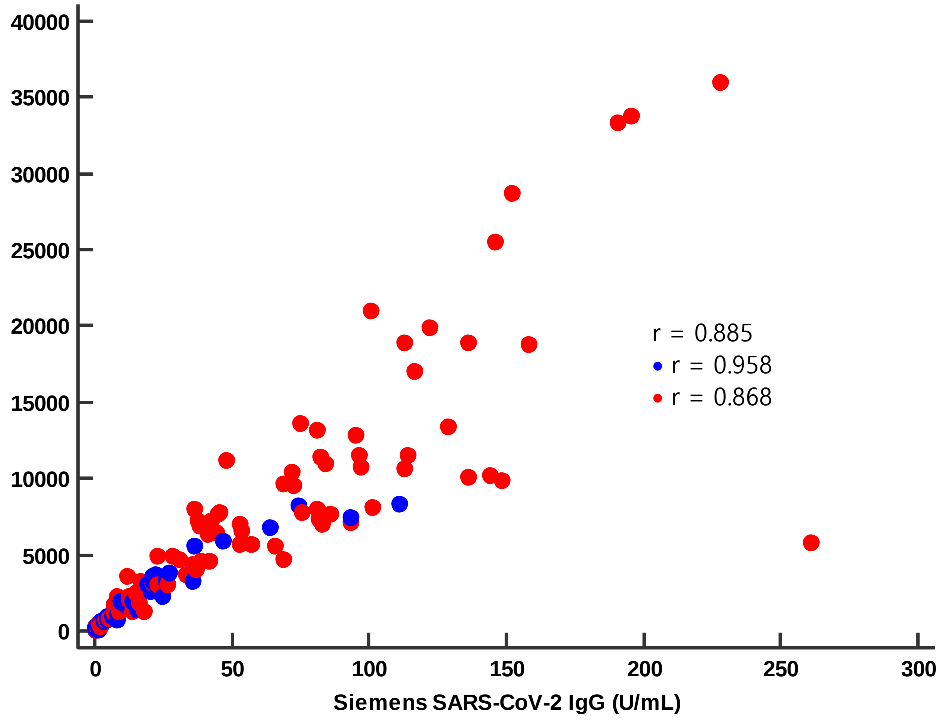
40000
35000
30000
25000
20000
15000
10000
5000
0
0
50
100
150
200
250
300
Siemens SARS-CoV-2 IgG (U/mL)
r = 0.885
r = 0.958
r = 0.868

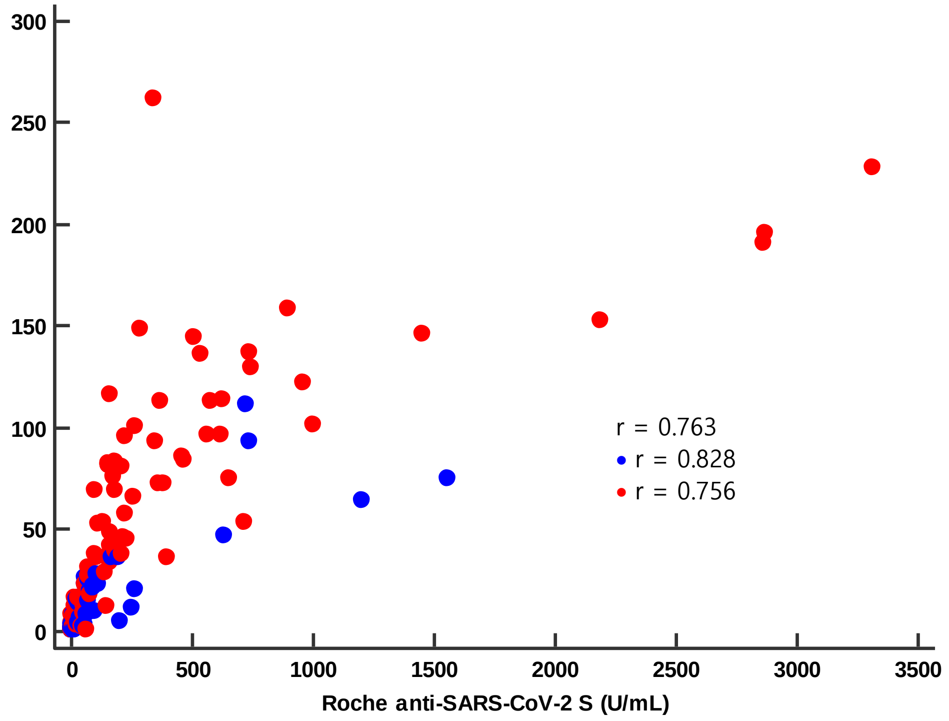


**Figure S2**. Spearman rank correlation between SARS-CoV-2 S protein antibody levels from three chemiluminescent assays.
